# Circuit Investigation of Social Interaction and Substance Use Disorder Using Miniscopes

**DOI:** 10.3389/fncir.2021.762441

**Published:** 2021-10-05

**Authors:** Nicholas J. Beacher, Kayden A. Washington, Craig T. Werner, Yan Zhang, Giovanni Barbera, Yun Li, Da-Ting Lin

**Affiliations:** ^1^Intramural Research Program, National Institute on Drug Abuse, National Institutes of Health, Baltimore, MD, United States; ^2^Department of Pharmacology and Physiology, Oklahoma State University Center for Health Sciences, Tulsa, OK, United States; ^3^Department of Zoology and Physiology, University of Wyoming, Laramie, WY, United States

**Keywords:** miniature fluorescence microscopy, miniscope, *in vivo* calcium imaging, longitudinal imaging, substance use disorder, social interaction

## Abstract

Substance use disorder (SUD) is comorbid with devastating health issues, social withdrawal, and isolation. Successful clinical treatments for SUD have used social interventions. Neurons can encode drug cues, and drug cues can trigger relapse. It is important to study how the activity in circuits and embedded cell types that encode drug cues develop in SUD. Exploring shared neurobiology between social interaction (SI) and SUD may explain why humans with access to social treatments still experience relapse. However, circuitry remains poorly characterized due to technical challenges in studying the complicated nature of SI and SUD. To understand the neural correlates of SI and SUD, it is important to: (1) identify cell types and circuits associated with SI and SUD, (2) record and manipulate neural activity encoding drug and social rewards over time, (3) monitor unrestrained animal behavior that allows reliable drug self-administration (SA) and SI. Miniaturized fluorescence microscopes (miniscopes) are ideally suited to meet these requirements. They can be used with gradient index (GRIN) lenses to image from deep brain structures implicated in SUD. Miniscopes can be combined with genetically encoded reporters to extract cell-type specific information. In this mini-review, we explore how miniscopes can be leveraged to uncover neural components of SI and SUD and advance potential therapeutic interventions.

## Introduction

Communicating or being around others, Social Interaction (SI), is crucial for health and wellness in humans (Hunt and Azrin, 1973; Kawachi and Berkman, 2001; Dishion and Stormshak, 2007), and animals (Council, 2010). People suffering from substance use disorder (SUD) oftentimes self-isolate from friends and family (Young A.M. et al., 2001), but fortunately, social interventions and group therapies promote drug abstinence (Hunt and Azrin, 1973). In preclinical models, animals self-administer (SA) SI over drugs of abuse in “choice” tasks (Venniro et al., 2019; Venniro and Shaham, 2020). However, people in recovery from SUD still experience relapse despite access to therapy (Nestler, 2002).

Similar reward-related brain areas underlie SI and SUD (Peoples and West, 1996; Young L.J. et al., 2001; Tang et al., 2005; Bergan et al., 2014; Holder et al., 2015; Manduca et al., 2016). It is critical to study the neurobiology underlying the therapeutic potential of SI in the treatment of SUD. First, it is important understand cell types and genetic information for potential molecular contributions to SI and SUD. Second, neurons can encode cues that are associated with drug rewards (Robinson and Berridge, 1993; Flagel et al., 2008) and drug craving and relapse can be triggered by drug cues in humans with SUD (Kennedy et al., 2013). Therefore, it is of great importance to monitor how neurons encode drug and social reward cues over extended periods of time and then directly manipulate them to determine their function. This need for *in vivo* observation of neural activity necessitates creating conditions to monitor unrestrained animal behavior that allows reliable drug SA and SI. However, circuitry underlying SI and SUD remains poorly characterized due to technical challenges and the complicated (and intertwined) nature of SI and SUD. Miniaturized fluorescence microscopes (miniscopes) are ideally suited to meet the outlined requirements Miniscopes in combination with gradient index (GRIN) lenses can record neurons in deep brain structures implicated in SI and SUD, and can be combined with optogenetic tools to manipulate neuronal activity and genetically encoded reporters to extract genetic and cell-type specific information.

The miniscope’s compact nature allows for the study of complex behaviors, such as SI and drug SA, in freely moving animals. In this mini-review we first discuss the genetic and hardware technologies that helped make the miniscope GRIN lens system ideal for working with *in vivo* animal models. We then explore how miniscopes can be leveraged to uncover the neurobiological components of SI and SUD in order to advance potential therapeutic interventions of SI for SUD.

## Parallel Advances in Calcium Imaging and Miniscope Development

**FIGURE 1:**
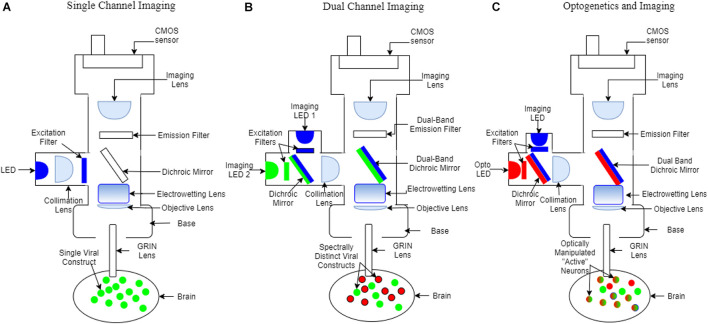
Schematic of miniscope configurations. Colors represent wavelengths (blue: ∼480 nm, green: ∼520 nm, red: ∼560 nm). **(A)** Single channel fluorescence miniscope and GRIN lens system for imaging virally transduced neurons, or other cells with a single fluorescence activity reporter. **(B)** Dual-color fluorescence miniscope and GRIN lens system for simultaneous imaging of multiple types or subtypes of neurons. Individual components that differ from the single channel miniscope include an additional excitation filter, dual-band dichroic mirrors and dual-band emission filters, two cell populations virally transduced with activity reporters with non-overlapping fluorescence (red and green), and, in this example, only a single CMOS detector which necessitates interleaving excitation and read-out of images. **(C)** Optogenetics miniscope used for simultaneous recording and manipulation of neurons within view of the GRIN lens. Differences from the dual-color miniscope include the use of a separate set of filters to allow simultaneous imaging and optogenetic inhibition and a single band emission filter. Strategies include transduction of opsins in connecting brain regions for optogenetic control and with activity reporters in the brain region being imaged. Neurons expressing opsins can be optogenetically stimulated (red) and neurons expressing activity reporters (green) can be imaged within the same experiment.

Calcium indicators have evolved from chemical dyes to genetically encoded proteins to provide insight on *in vivo* neural activity (Rose et al., 2014). Genetically encoded calcium indicators (GECIs) have allowed cell-specific targeting of neurons to measure transient increases in Ca^2+^ associated with neuronal spiking (Tian et al., 2009; Chen et al., 2013; Gerfen et al., 2013; Rose et al., 2014; Zhang et al., 2020). Active neurons fire action potentials and result in transient increases in intracellular Ca^2+^ which upon binding to a GECI can increase its fluorescence intensity (Nakai et al., 2001; Tallini et al., 2006; Akerboom et al., 2012; Förster et al., 2018; Yang et al., 2018). Therefore, individual neuronal activity can be readily monitored through fluorescence changes of the GECI. In addition, the development of head mounted widefield miniaturized fluorescence microscopes (miniscopes) paired with Gradient Index (GRIN) lens implantation, enables deep brain *in vivo* Ca^2+^ imaging and analysis of neural mechanisms in awake and freely behaving animals (Ghosh et al., 2011; Markowitz et al., 2015; Barbera et al., 2016, 2019; Cai et al., 2016; Okuyama et al., 2016; Klaus et al., 2017; Chen et al., 2018; Jimenez et al., 2018; Liang et al., 2018; Werner et al., 2019; Zhang et al., 2019; de Groot et al., 2020; Shuman et al., 2020; Figure 1).

Through the implanted GRIN lens, fluorescent signals emitted by neurons are collected by the miniscope (Leiner and Prescott, 1983; Levene et al., 2004; Zhang et al., 2019; Laing et al., 2021). This miniscope and GRIN lens system allows for exploring a variety of aspects of neural activity from individual neurons and neural ensembles (Bollimunta et al., 2021). Some of these aspects include identifying neuronal subtypes and observing microcircuits that encode behavior. The identified circuits can then be manipulated through optogenetics to determine function (Kondabolu et al., 2015; Martín et al., 2015; Barbera et al., 2016; Heinsbroek et al., 2017; Natsubori et al., 2017; Parker et al., 2018).

Recent advances have been made to the miniscope procedure, such as biologically safe coating on the GRIN lens (Kyogoku et al., 1990; Yang et al., 2019) which would otherwise leak toxic compounds and cause neurodegeneration near the site of the lens implantation (Table 1). Higher precision surgical tools have also allowed for a greater level of sustainability and survivability post-surgery (Liang et al., 2019). With decreased lens toxicity and reduced surgically induced inflammation researchers can follow the activity of neurons over time using miniscopes and image healthier tissue. Furthermore, increased animal mobility during miniscope imaging has been achieved with the advent of motorized commutators (Coward and O’Boyle, 1979; Fee and Leonardo, 2001; Barbera et al., 2020) and wire-free miniscopes (Barbera et al., 2019; Shuman et al., 2020; Table 1). The advent of deep learning algorithms to track animal behavior has helped better align neural activity with behavior in individual and groups of animals, which is important for the study of SI and SUD. For example, behavior can be analyzed using deep learning software (Hong et al., 2015; Mathis et al., 2018; Mosser et al., 2021) such as DeepLabCut (Mathis et al., 2018; Nath et al., 2019; Table 1). Dual color miniscopes can record from two cell types simultaneously within the same animal, which provides a powerful tool to uncover spatiotemporal dynamics of activity in multiple cell subpopulations that are active during SI and in SUD (Jacob et al., 2018; Leman et al., 2018; Figure 1). An Electrowetting Lens (Kuiper and Hendriks, 2004; Ren and Wu, 2007) is able to remotely adjust focus between neurons across different focal planes and can also be incorporated into miniscopes (Hayashi et al., 2021; Figure 1). In combination these advancements have greatly facilitated uncovering the spatiotemporal dynamics of neural activity during behavior.

**TABLE 1:** Summary of miniscope benefits.

**Benefit**	**Examples**	**References**
1. Identification of Cell Types	GABAergic D1- expressing Neurons: • Drd1-iCre	GABAergic D1- expressing neurons: • Pettibone et al., 2019
	GABAergic D2- expressing Neurons: • Adora2-iCre	GABAergic D2- expressing neurons: • Pettibone et al., 2019
	Glutamatergic Neurons: • Vglut2-iCre	Glutamatergic Neurons: • Heinsbroek et al., 2020
	GABAergic Neurons: • Vgat-iCre	GABAergic Neurons: • Heinsbroek et al., 2020
2. Longitudinal Imaging in areas relevant to SI and SUD and Manipulation of Ensembles	Longitudinal Imaging of ensembles: • Nucleus Accumbens • Prefrontal Cortex • Hippocampus	Longitudinal Imaging of ensembles • Zhang et al., 2019 • Liang et al., 2018; Kingsbury et al., 2019; Kingsbury et al., 2020 • Jimenez et al., 2018
	Optogenetics Capable Miniscopes	Optogenetics Capable Miniscopes • Stamatakis et al., 2018; de Groot et al., 2020
3. Behavioral Tools and Analysis Software	Wire-free Miniscopes:	Wire-free Miniscopes: • Barbera et al., 2019; Shuman et al., 2020
	Motorized Commutator:	Motorized Commutator: • Coward and O’Boyle, 1979; Fee and Leonardo, 2001; Barbera et al., 2020
	GRIN lens coating to avert impact on tissue health:	GRIN lens coating to avert impact on tissue health: • Kyogoku et al., 1990; Yang et al., 2019
	Deep Learning Behavioral Analyses:	Deep Learning Behavioral Analysis: • Hong et al., 2015; Mathis et al., 2018; Mosser et al., 2021

## Benefits of Using Miniscopes in Animal Models

### Identifying Genetic Components and/or Specific Cell Subtypes

The first step in dissecting the underlying neurobiological contributions associated with SI and SUD is to determine the exact types of cells that are being recorded. For example, the nucleus accumbens (NAc) is an area within the dopamine system implicated for SI (Manduca et al., 2016), and drug reward (Peoples and West, 1996). There is evidence that NAc MSNs are implicated in reward-associated cue presentation (Owesson-White et al., 2016) and cued reward seeking of alcohol (Valyear et al., 2020). NAc neurons also encode the lever press (Peoples and West, 1996; Owesson-White et al., 2016), reward approach (Valyear et al., 2020), and reward consumption (Nicola et al., 2004; Stamos, 2019). The NAc is composed of different types of cells; the majority medium spiny neurons (MSNs) (Kupchik and Kalivas, 2017) with the remainder primarily composed of other cells such as fast spiking interneurons (FSIs) (Schall et al., 2021). Some *in vivo* recording methods, such as electrophysiology, distinguish differential cell types by way of differential electrical patterns (Schall et al., 2021), including using waveform patterns to differentiate MSNs from FSIs (Berke et al., 2004; Morra et al., 2010; Sun and Laviolette, 2014). Many advancements in neuroscience were performed with *in vivo* electrophysiology. For example, MSNs contribute differentially *-* some increase in firing prior to a drug stimulus while others decrease (Peoples and West, 1996; Peoples et al., 1998). We now know that cellular subtypes of MSNs exist, and that NAc MSNs contain dopamine D1 or D2 receptor subtypes with distinct projections (Clark and White, 1987; Ikemoto et al., 1997; Kupchik et al., 2015). These subtypes are thought to contribute to processing motivation differentially. For example, NAc D1-MSNs encode reward (Tran et al., 2005; Lobo et al., 2010) while NAc D2-MSNs encode aversion (Danjo et al., 2014). In addition, it has been shown that NAc D2-MSNs are involved in reward (Kupchik et al., 2015; Soares-Cunha et al., 2016; Cole et al., 2018) or a combinatory effect of D1- and D2-MSNs encode reward motivated behavior (Ikemoto et al., 1997; Manduca et al., 2016).

*In vivo* electrophysiology recordings have limitations to readily separate D1- and D2- MSNs. Miniscopes, by contrast, provide a means to distinguish D1- and D2- MSNs. Researchers using miniscopes can take full advantage of modern advances in newly available transgenic animals (Pettibone et al., 2019; Heinsbroek et al., 2020; Table 1). By combining transgenic animals with Cre-dependent GECIs it is possible to target specific cell populations in the NAc and assess their contribution to SI and SUD (Zhang et al., 2019). With dual color miniscopes (Aharoni and Hoogland, 2019) being released, two cell populations can be imaged concurrently using GECIs with different excitation wavelengths (Park K. et al., 2021; Figure 1). This makes it possible to simultaneously dissect the contributions of multiple cell types from the same subject undergoing SI and drug SA. The amygdala, which is involved in SI (Bergan et al., 2014) and SUD (Holder et al., 2015) can be monitored with the same approach. GABAergic Amygdala neurons promote SI while glutamatergic neurons promoted self-grooming (no SI behaviors) (Hong et al., 2014). This GABAergic and glutamatergic dichotomy can also be applied to ventral pallidum (VP) (Heinsbroek et al., 2020) which is also implicated in drug (Tang et al., 2005) and social reward (Young L.J. et al., 2001). Another example of differential subtype involvement is in the prefrontal cortex (PFC) during social fear. Local microcircuits within PFC disinhibit other subtypes of inhibitory interneurons in order to gate social fear behavior (Xu et al., 2019). Using the miniscope to understand distinct contributions of cell subtypes such as these would be a major step forward in SI and SUD research (Table 1).

### Track Neural Activity Over Extended Periods of Time and Directly Manipulate Circuits

The intricacies of circuitry involved in drug and social rewards are vast but critically important to study. It is also known that different rewards (such as food vs. drugs of abuse) may not necessarily be encoded by the same neurons (Martin-Fardon et al., 2018). Currently, we do not yet know how individual neurons or neural ensembles encode the choice between drugs vs. social reward (Venniro et al., 2019; Venniro and Shaham, 2020) or experience drug SA followed by social SA on different days. Do the same or distinct neurons and neural ensembles encode both drug and social SA? In the literature, animals can self-administer the opportunity to act aggressively to a smaller submissive animal (Golden et al., 2019) but how would these same neurons encode the opportunity to self-administer a more positively valanced SI (Venniro et al., 2018; Venniro and Shaham, 2020)? Furthermore, if a negative SI or drug situation is experienced (such as in the synchrony with pain or distress), how are neurons reorganized in response to these novel contexts? The miniscope, in combination with a GRIN lens, uses *in vivo* fluorescence imaging to track neuron activity over time during unrestrained behaviors, which provides an opportunity to study how the brain encodes drug SA and/or SI.

Miniscopes have been used to study neural activity in brain regions such as the NAc (Zhang et al., 2019) and PFC (Liang et al., 2018; Kingsbury et al., 2019, 2020; Table 1). These two regions are implicated in SI and SUD and thus are important targets of cellular resolution imaging (Peoples and West, 1996; Kalivas et al., 2005; Quintero, 2013; Manduca et al., 2016; Liang et al., 2018; Kingsbury et al., 2019, 2020; Xu et al., 2019). It is also important to consider imaging regions that project to the NAc and PFC. For example, the Hippocampus has been studied using miniscopes (Jimenez et al., 2018) SI memories are encoded in Hippocampus (Liu et al., 2019; Wu et al., 2020) and morphine withdrawal is associated with hyperactive glutamatergic uptake in the Hippocampus (Xu et al., 2003). The ventral tegmental area (VTA) is another region of interest involved in drug (Sun et al., 2005) and social reward (Hung et al., 2017).

Beyond the identification of cell type and cell “function,” another benefit of the miniscope is the possibility for subsequent manipulation of the involved circuits at fine temporal resolutions using optogenetics, which can be used in addition to imaging (Stamatakis et al., 2018; de Groot et al., 2020; Figure 1; Table 1). The NAc and PFC circuit is strengthened during incubation of drug craving (Luís et al., 2017) and involved in social recognition (Park G. et al., 2021) which is impaired following social isolation. Through imaging, cellular ensemble activity can be correlated with behavior, but manipulation of ensembles is required to dissect functional roles. For example, would disrupting the PFC to NAc circuitry reduce incubation of drug craving? Similarly, would exciting this pathway overcome the impairment induced by social isolation? Neuroscientists can utilize tools such as total ablations of pathways (Kelly et al., 1975) but these are permanent changes that take place over days or weeks of time, lack temporal control, and affect other behaviors. Additionally, circuitry can be altered *via* chemogenic manipulations (Mahler and Aston-Jones, 2018), however, this approach has far less temporal control than optogenetics (taking time to onset and offset). These problems can be overcome with an optogenetics capable miniscope by e.g., using one channel to perform optogenetic activation or silencing of projection populations, or surrounding cells, while simultaneously imaging with the second channel (Stamatakis et al., 2018; de Groot et al., 2020; Figure 1; Table 1). In this scenario, optogenetic manipulations often consist of cell-specific viral transduction of cells with opsins that are then excited at wavelengths that can be separated from the excitation and emission wavelengths of the imaged fluorophore (Fenno et al., 2011). Some other technologies require a simultaneous implant of an optical fiber along with the recording device for optogenetic manipulations (Kravitz et al., 2013). This fiber-coupled technique can be used in conjunction with miniscopes which may lack the second “built-in” optogenetic channel (Hong et al., 2014).

### Monitoring Unrestrained Animal Behavior That Allows Reliable Drug Self-Administration and Social Interaction

One of the major advantages of miniscope imaging is the ability to study activity in awake, freely behaving animals. The miniscope can be mounted on the head of an animal with a light tether which allows the animal to perform behaviors naturally. Typical SI models require the need for active socialization (Rodriguiz et al., 2004; Manduca et al., 2016; Liang et al., 2018; Venniro et al., 2018, 2019; Golden et al., 2019; Venniro and Shaham, 2020), and the compact nature of the miniscope allows for the animal to freely explore it’s environment. Using miniscopes, the animal is tethered to a freely rotating swivel which can be counterbalanced to reduce the overall weight on the animal (Zhang et al., 2019). This mechanism is similar to successful techniques used for other *in vivo* recordings such as electrophysiology (Fabbricatore et al., 2009). Wire-free miniscopes have also been developed (Barbera et al., 2019; Shuman et al., 2020) which can allow for study of SI in larger groups that would otherwise tangle and destroy wired systems. Moreover, advancements in behavior analysis (Hong et al., 2015; Mosser et al., 2021) enable neural activity imaged with miniscopes to be correlated with discrete phases of behavior. Studying the emergence of how specific subtypes of neurons encode various components of behavioral sequences of SI and SUD is critical to understanding overlapping neurobiology and to preventing drug relapse.

Given the ability of an animal to move freely, miniscopes allow for the implementation of more suitable behavioral paradigms that are relevant to SUD and SI. For example, SI can been modeled in many ways, including SI “choice” tasks (Venniro et al., 2018, 2019; Venniro and Shaham, 2020), sexual cue preference tasks (Bergan et al., 2014; Yao et al., 2017), and social fear (Toth and Neumann, 2013; Gutzeit et al., 2020). Similarly, SUD can be modeled in different ways; long-access SA (Roth and Carroll, 2004; Kitamura et al., 2006; Shepard et al., 2006; Rogers et al., 2008; Algallal et al., 2020; Klein et al., 2020) intermittent-access SA (Kawa et al., 2016; Nicolas et al., 2019; Algallal et al., 2020), and short-access SA (Roth and Carroll, 2004; Kitamura et al., 2006). Studying how neurons encode behaviors expressed in these different behavioral paradigms is critically important for increasing the translational validity of results to improving the human condition.

## Discussion

The use of SI in the treatment of SUD is a promising approach to help those suffering from SUD. Understanding the neurobiology of SI and SUD is crucial to understand why some people with SUD and access to treatment still suffer from drug relapse. In this review, we focused on three important aspects of studying the neurobiology of SI and SUD: identification of cell type and genetic influences, longitudinal imaging of individual neurons and neural ensembles with the potential for manipulation, and *in vivo* animal models. Dissecting the individual contributions of neural mechanisms requires temporally precise analysis. A limitation of calcium imaging is the performance of calcium sensors (Storace et al., 2015) where changes in calcium act a proxy of voltage fluctuations (Wachowiak et al., 2013). Furthermore, there are limitations of the imaging sensors used in miniscopes. However, genetically encoded voltage indicators (GEVIs) (Bando et al., 2019) and improved image sensors (Marshall et al., 2016; Juneau et al., 2018) offer a new horizon for increasing the temporal precision of miniscopes.

Through technological advancements in indicators, sensors, and imaging hardware, our understanding of the neurobiology underlying SI and SUD can be expanded. New analysis methods allow for the extraction of new information from imaging datasets, and miniscope imaging can be merged with deep learning software to extract cell type and circuit functions involved in SI- and SUD-related behaviors.

In summary, miniscopes allow for identification of distinct cell types. Miniscopes also allow for tracking the activity of neurons over extended periods of time and directly manipulating those microcircuits. Finally, miniscopes enable conditions to monitor unrestrained animal behavior that allows reliable SA. Together, miniscope imaging is a fantastic tool to identify underlying neurobiological functions of SI and SUD.

## Author Contributions

All authors prepared and edited the manuscript. NJB, KAW, and D-TL wrote the manuscript.

## Conflict of Interest

The authors declare that the research was conducted in the absence of any commercial or financial relationships that could be construed as a potential conflict of interest.

## Publisher’s Note

All claims expressed in this article are solely those of the authors and do not necessarily represent those of their affiliated organizations, or those of the publisher, the editors and the reviewers. Any product that may be evaluated in this article, or claim that may be made by its manufacturer, is not guaranteed or endorsed by the publisher.
